# Dance and Somatic-Informed Movement in an Acute Inpatient Stroke Unit

**DOI:** 10.3390/medicina61060966

**Published:** 2025-05-23

**Authors:** Lucie Beaudry, Céline Odier, Sylvie Fortin

**Affiliations:** 1 Department of Dance, Université du Québec à Montréal, Montréal, QC H2L 1H4, Canada; fortin.sylvie@uqam.ca; 2 Centre for Interdisciplinary Research in Rehabilitation (CRIR) of Greater Montreal, Montréal, QC H3S 1M9, Canada; 3 Department of Neurosciences, Université de Montréal, Montréal, QC H3K 1M9, Canada; celine.odier@umontreal.ca; 4 Centre de Recherche du Centre Hospitalier de l’Université de Montréal (CRCHUM), Montréal, QC H2X 0A9, Canada

**Keywords:** somatic-informed movement practice (SIMP), Feldenkrais Method^®^, dance, stroke acute care, qualitative study, critical incident technique

## Abstract

*Background and Objectives*: Stroke units rely on interdisciplinary teams. Professionals with complementary alternative practices may join the team since such approaches are increasingly supporting the stroke recovery process. The aim of this study was to develop a better understanding of how a dance and somatic-informed movement intervention could be utilized in an inpatient setting as an adjunct to post-stroke therapy. We sought to identify (1) what knowledge we could draw on to develop the content and pedagogy for the intervention, (2) what helped/hindered the intervention aimed at functional recovery, as perceived by the practitioner-researchers, and (3) the relationships experienced with the various stakeholders. *Materials and Methods:* This exploratory qualitative study used the enhanced critical incident technique to collect retrospective self-report data from two practitioner-researchers engaged in delivering the intervention over two months. The data underwent thematic analysis. Patients (n = 6) in a stroke unit were selected within ≤72 h of hospital admission. The intervention was conducted four to six times a week until the vascular neurologist (co-researcher) authorized their transfer to a rehabilitation hospital. *Results*: The intervention evolved from crafting content and pedagogy at the intersection of different areas of knowledge (dance, somatics, neuroscience, and stroke). It was based on active, assisted, and passive movements. Verbal, tactile, visual, and imaginary inputs used to enhance body awareness were perceived as potentially helping patients recover some range of motion, quality of movements, and voluntary movement control, and fostering calmness and motivation. The intervention was well received by stakeholders. *Conclusions*: Dance and somatic-informed movement can be a complementary therapy in stroke units, although it requires a delicate juggling of time allocation within the interdisciplinary team. Further studies should be conducted with a larger number of patients and different practitioners. Collaboration between qualitative and quantitative researchers is needed to make a robust case for such interventions.

## 1. Introduction

Stroke is a leading cause of disability worldwide [1] that can cause lasting brain damage and long-term disability. Stroke best practices suggest that early and intensive rehabilitation care for patients in both the acute and subacute phases of stroke helps to optimize their recovery [2]. This is why stroke care units seek to provide rehabilitation therapy as soon as patients are medically stable and able to participate. The acute phase of stroke generally refers to the first two weeks of inpatient treatment, while 3–11 weeks post-stroke is termed the subacute phase of stroke [3]. Stroke units traditionally rely on an interdisciplinary team including vascular neurologists, physicians, nurses, occupational therapists, physical therapists, speech therapists, psychologists, social workers, and dietitians. However, other professionals with complementary alternative practices may join the team since non-pharmacological approaches are gaining attention as a means of supporting the wider stroke recovery process alongside therapies focused on functional recovery [4].

Somatic-informed movement practice (SIMP) is part of these initiatives in health settings [5]. Somatics is a field of study and practice based on the proprioceptive and kinesthetic experience of movement. It draws on the notion of soma, i.e., “the body as perceived from within” [6] (p. 4). In the broad field of somatics, SIMP is a specific area of dance practice “foregrounding an approach to the body and movement that uses improvisation, sensory awareness, imagination and touch” [5] (p. 290). Tufnell [7] points out that it “is not ‘dance’ in the conventional sense, but rather communication through movement and the body” (p. 105).

Dance studies with groups of stroke patients have been conducted during different phases of stroke, whether in hospitals [8,9], outpatient clinics [10], community settings [11], or university settings [12]. Documented benefits mainly concern mobility and gait parameters, balance, emotional functions, and social interactions. Calil et al. [13] used a somatic approach to dance, focusing on the dancer’s internal sensations. They concluded that dance requires awakened sensations that give rise to emotions, promoting muscular relaxation and making movement more efficient. Only one of these studies with stroke survivors was conducted in an acute hospital setting. Bungay et al. [8] offered a dance program to groups including elderly stroke survivors.

Beaudry et al. [14,15,16] conducted three interrelated studies using dance in subacute post-stroke rehabilitation, as well as strategies inspired by the Feldenkrais Method^®^ (FM), one of the most established somatic approaches. They reported benefits in terms of mobility, emotional functions, motivation, social interactions, and perception of effort.

The FM offers a complementary approach to perceptual-motor learning [17] by asking people to engage with sensations and perceptions embedded in detailed, guided movement explorations [18]. It seeks to improve deficient connections between the motor cortex and body movement. The method offers a group and individual modality called, respectively, Awareness Through Movement (ATM) and Functional Integration (FI), which are derived from the same theoretical basis [19]. Both rely on fundamental motor patterns (e.g., extensions, flexions) and activities of daily living (e.g., turning around, moving from sitting to standing position). The FM is usually taught in one-hour classes, where people are encouraged to move slowly and comfortably while paying attention to movement details. This method has been the subject of health-related studies.

Studies using the FM with stroke patients have shown that it can induce an improvement in balance [20], reduce post-stroke spasticity [21], and have a positive impact on self-awareness and attitude [22]. Feldenkrais [23] himself documented the motor learning of a woman who had a stroke, and the benefits of FI on her attention, perception, and cognition. According to Doidge [24], this case report demonstrated the application of neuroplasticity theory long before task-based functional MRI demonstrated the brain’s ability to modify its structural and functional organization following brain damage.

In our literature review, we found no studies examining a combination of somatics and dance in stroke care units. Describing the intervention as such is, therefore, a logical first step in providing essential information likely to support potential benefits.

No ‘best practice guides’ exist for such movement sessions. The aim of this exploratory study was thus to investigate how dance and somatic-informed movement practice (SIMP) could be used as a complementary intervention for patients in a stroke care unit. This article presents the practitioner-researchers’ fieldwork attempts at “dancing with the brain”, an expression used by Moshe Feldenkrais, although what he refers to as dancing had “no stereotyped technique to apply ready-made to everyone” [24] (p. 179). Given the uniqueness of the research project, the following questions guided our encounters with the patients and interdisciplinary team at the hospital: (1) What knowledge can we draw on to develop the content and pedagogy for our intervention? (2) What could help or hinder our intervention in terms of improving the patients’ functionality? (3) What relationships can we develop with the medical team, the patients, and their relatives?

## 2. Materials and Methods 

### 2.1. Qualitative Research

To answer the above research questions, we conducted qualitative research, namely a collaborative autoethnography. It consists of two or more persons who analyze and report their occupational and emotional experiences as professionals working in healthcare systems [25]. We specifically used the critical incident technique (CIT), which is “a qualitative research tool that is frequently used in health services research to explore what helps or hinders in providing good quality care or achieving satisfaction with care provision” [26] (p. 1065). The CIT originated from Flanagan [27], who developed procedures for collecting direct observations of human behaviour. Since then, researchers have adjusted their focus from direct observation to include retrospective self-report, i.e., “investigation of psychological constructs and of experiences, perceptions, and beliefs” [28] (p. 745), which is now referred to as the enhanced critical incident technique (ECIT). This shift reflects an epistemological stance that embraces the dual roles we had (LB and SF) as practitioner-researchers engaged in planning and delivering the intervention, as well as collecting and analyzing data. The self-generated observations of ourselves and the patients we worked with run counter to the hegemonic observer–object relationship at the core of the positivistic scientific method.

### 2.2. Data Collection and Analysis

The data collected consisted of (1) detailed descriptions of the critical incidents we perceived as helping or hindering the intervention, (2) the reasons why we perceived them as helping or hindering, (3) the logics of action, i.e., knowledge base or intuitions that supported our actions, (4) descriptions of the context, including relationships with the different persons involved, and (5) a wish list about the context and about how the next individual movement session should unfold. Data were collected through a questionnaire (see Table 1) completed by the practitioner-researchers as soon as possible after their sessions. It was also collected from reflexive research journals in which a written account was kept of (i) the regular debriefing conversations between the two practitioner-researchers and of (ii) the weekly interdisciplinary meetings at which each patient’s conditions were discussed from the different disciplinary perspectives, including that of the third author-researcher taking part in this project as a vascular neurologist.

In line with the principles of thematic analysis [29], we read the entire corpus of data (critical incidents) to start the coding process and identified categories of information relevant to our research questions. The identified categories then revealed overarching themes. The categories and themes that emerged were discussed by the research team until consensus was reached. Data saturation was monitored to determine when no new codes were emerging from the data.

In the end, this process turned out to be only partially satisfactory because it did not reveal the logic of action, which could be ascertained solely by showing the day-by-day progression of the intervention. Thus, inspired by Richardson and St-Pierre [30], we wove the fieldwork results into a concise and ongoing narrative of our encounters with a selected sub-corpus of patients and sessions, specifically for an academic and medical audience. To ensure the trustworthiness of our findings, we applied typical key criteria of credibility, transferability, dependability, and confirmability [31].

### 2.3. Intervention Procedures

This study was conducted in a metropolitan public hospital in Canada that receives about 400 stroke admissions per year. The number of patients we worked with depended on the number of stroke admissions during a predetermined period of two months devoted to data collection. Patients were selected within 72 h of hospital admission. Inclusion criteria were moderate to severe acute ischemic or hemorrhagic stroke according to the National Institutes of Health Stroke Scale [32] (NIHSS initial > 15), with non-significant improvement in the first 24–48 h (NIHSS improvement < 2 points) after admission. Exclusion criteria were aggressiveness, absence of eye contact, lacunar stroke, those who had palliative care planned within 2 weeks, and no restrictions according to neurological deficits (including somnolence).

It is customary in dance research to present information such as the nature of a typical warm-up and of a choreographed or improvised dance. Because of the changing physical, cognitive, and emotional states of the patients in our study, it was impossible to plan in detail the content of the individual movement sessions upstream. Each day, we had in front of us a patient for whom we needed to decide what action to take on the spot or almost on the spot. We were constantly observing and interacting, using our hands or words to invite the patient to move different body parts, and reassessing our intervention continually based on the patient’s verbal and nonverbal cues. As portions of individual movement sessions will be described in detail in Section 3, it is sufficient to say here that the content relied on the adaptation of ATM [33] and FI [34].

This study had received prior ethics approval from the Research Ethics Committee of the University of Montreal Hospital Centre (14.256) and from the Institutional Committee on Ethics in Human Research of the Université du Québec à Montréal (A-140017). Written informed consent was obtained from each patient as language and cognitive functions permitted, if not, from a family member or close friend. Pseudonyms are used in this article. All consents were collected by the vascular neurologist involved in the research after giving a one-on-one explanation of the project to one of these individuals.

The dance and somatic-informed movement intervention was taught by two university researchers who signed a confidentiality agreement. They were experienced dance teachers and somatic practitioners working with a wide range of populations. The vascular neurologist, co-researcher, was specialized in acute stroke care and stroke rehabilitation.

## 3. Results

We worked with six patients (mean age 72.2 years ± 9.2, range 58–83), including four women and two men, over a period of 50 days. Four patients presented with ischemic stroke, one of them with hemorrhagic transformation. One patient had a left stroke, one a right stroke, one a brainstem stroke, and, lastly, a spinal cord stroke. Two had hemorrhagic strokes (one left, one right). Median NIHSS (stroke severity score) was 11 (7–27) at the time of inclusion in the study. Two patients had severe aphasia, and one had a language barrier. Two patients had early spasticity. No patient was intubated or in the intensive care unit. Most individual movement sessions took place at the patient’s bedside four to six times a week, at a time that did not interfere with medical examination and therapies. They began, on average, 3.8 days (±2, range 1–7) after hospital admission and lasted until patients were transferred to a rehabilitation hospital, usually within two weeks. Each patient was followed by the same practitioner-researcher, who received a description of the patient’s condition from the vascular neurologist.

We offered an average of 6.5 sessions per patient (±3.5, range 2–12) over an interval of 10.5 days for each patient (±5.05, range 3–17). The average session duration was 41 min (range 15–80), depending on the patient’s capacities. The average number of hours spent with each patient was 4 h 30 min (±3 h, range 1.16 h–8.5 h). However, as we sometimes had to deal with pre-session waiting times, the average time spent on site for each patient was 5 h 17 min (±3.91 h, range 1.16 h–11.58 h).

Through our analysis of the qualitative data, we identified the themes associated with our research questions, which we categorized as K (knowledge), H (help), and R (relationships). Firstly, we identified the main areas of knowledge (K) on which we drew to develop the intervention, namely dance, somatics, neuroscience, and stroke (see Table 2). Secondly, we identified the main themes associated with the factors that helped (H) the intervention in terms of improving patients’ functionality. Despite a rigorous analysis of our data, we did not identify any elements that could have hindered our intervention and thus potentially have adversely affected the patients’ recovery process. Presumably, this is because we reacted quickly to elements that could be improved. Our data analysis identified a range of verbal, tactile, visual, and imaginary inputs that contributed to the intervention. The verbal inputs were information, instructions, and questions addressed to the patients. The tactile inputs were different types of touch, such as inductive, supportive, and reflective, used, respectively, to guide the persons in motion, support their movements, or focus their attention. The visual inputs consisted of movement demonstrations and eye contact (use of gaze to connect and to motivate). The imaginary inputs were essentially invitations to visualize difficult movements. We used active, assisted, and passive movements in the intervention, in an effortless (if active or assisted), gentle, progressive, repetitive, rhythmic, and slow manner. The notion of aesthetic environment proved to enhance the atmosphere of the intervention, whether through music or by introducing a qualitative and affective experience of movement. Lastly, our analysis enabled us to identify the relationships (R) we developed with the various people involved in the intervention, interactions that were rooted in a spirit of open-mindedness, curiosity, trust, dialogue, and collaboration. Table 2 below shows all the categories and themes that emerged from the thematic analysis, while an extract from the coding process is presented as a supplement at the end of the text (see Appendix A).

To keep the results presentation to a reasonable length, in the following sub-sections, we concentrate on two of the six patients who differed in terms of gender, sequelae, and functionality. We present a sub-corpus of their individual sessions that is representative of our encounters with each of them (Table 3 and Table 4) to substantiate the results. In the left-hand column, we use the present tense to reflect our immediate, embodied experiences with the patients; in the middle column, we use italics to report our theoretical and self-critical reflexivity; in the right-hand column, we present the themes associated with our research questions, which we categorize as K (knowledge), H (help), or R (relationships).

Our exploratory study did not aim to identify intervention benefits. However, when examining what could help the intervention in terms of patient functionality, data analysis revealed that our intervention had potential, especially in terms of (1) increasing the patient’s range of motion and quality of movements, presumably through better body part coordination, (2) inducing calmness in the patient’s state through strategies that encouraged letting go of unnecessary tension, (3) yielding gains in voluntary movement control (sometimes over involuntary movement), probably by providing detailed sensory information, and (4) increasing motivation to pursue rehabilitation, likely related to the music and sensory approach used. Although not results per se, these perceived benefits for patient functionality (F) are presented in the right-hand column of Table 3 and Table 4.

The following sub-sections are preceded by a brief presentation of each of the two patients.

### 3.1. Mr. K

Mr. K, a 73-year-old French-Canadian, lived alone but often stayed at his partner Helen’s place, where he suffered an ischemic stroke that affected the left hemisphere of his brain. On arrival at the hospital, he underwent thrombolysis, but without recanalization of the cervical carotid artery. The brain was well-irrigated by other arterial routes, and there were no contraindications to movement. His right side was plegic (lower and upper limbs), and he was aphasic. His NIHSS was 15 at the time of inclusion in the study. Before his stroke, he was physically active. I (SF) first met Mr. K about 24 h after his admission to hospital. I met him 12 times over 17 days for a total of 8 h 30 min of individual movement session time (average of 42.5 min/session).

**Table 3:** Mr. K—narrative based on a selected sub-corpus of sessions.

Session 1—14 January (50 min)		
Immediate and embodied experiences	*Self-critical theoretical reflection*	Themes and categories
After a brief introduction, I tell Mr. K [who is] lying in bed that what we are going to do together “is to put some movement back into his body” and that I am going to put my hand on his head to roll it very gently from side to side, which I did, humming a gentle rhythm. I notice a little movement to the left and a lot of resistance to the right.	*I stand to his left and lower the bedpost to make sure that he can see and hear me, and that he understands my instructions. I pace the action because I know the cognitive benefits of rhythm for the brain* [35].	(K) Somatics, stroke (H) Inductive and supportive touch, passive and assisted movement, rhythm, gentleness, verbal information (F) Range of motion
I ask him to do the movement with me. At first, he moves fast, puts in a lot of effort, and his head barely rolls to the right. I tell him that we will repeat more slowly and gently to feel the movement better and use only the minimum muscular effort required. Gradually, he slows down, and my hand can feel that he is not overly contracting his muscles.	*Both the sound and cadence of my voice, and the touch of my hand on his forehead, encourage gentleness and slowness. The FM is based, among other things, on Weber–Fechner’s law* [36]*, which refers to the minimum intensity of a stimulus required for one to detect its presence.*	(K) Neuroscience, somatics (H) Supportive touch, active movement, repetition, slowness, gentleness, rhythm, effortless, verbal information, and instruction (F) Tension release
I invite Helen, his partner, to stand on the right side of the bed, and ask Mr. K to slowly and rhythmically pronounce her first name with me and her when he tries to turn his head to the right, and my first name when he turns his head to the left (I support the rolling movement with my hand on his forehead). Mr. K cannot speak, but I invite him to say our names in his head as his head moves from side to side. His eyes are blurry when he turns to the right. I then ask Helen to position herself at an angle that allows good eye contact. We continue in this way for several repetitions. Gradually, he succeeds in establishing clear eye contact with his head rolling more easily to the plegic side. He relaxes. He smiles at us.	*On the spur of the moment, I asked Mr. K’s partner to participate because every bodily movement is motivated by desire. Mr. K became motivated by a meaningful visual and sound stimulus. I perceived this proposal as helping, at both an affective and a motor level. I chose this movement to start with because “eye movements affect muscular tone of the neck and the rest of the body”* [36] *(p. 200). It is often used in the FM, which relies on motor development principles.*	(K) Neuroscience, somatics (H) Supportive touch, active movement, repetition, gaze, rhythm (R) Collaboration (F) Tension release, body part coordination, motivation
**Session 6—21 January (45 min)**		
We go into the physiotherapy room. Through the window, we can see a splendid sunset. I mention, “It’s so beautiful; let’s take two minutes to appreciate the many shades of pink”. His partner joins us, puts a hand on his shoulder. We all feel moved as we admire the sunset in silence.	*One of the benefits of dance as an aesthetic activity in health is that it helps to regulate emotions* [37]*. In the FM, taking time to create an atmosphere conducive to perceptual learning is foremost. Doidge* [24] *explains that with the FM, the injured brain, which has been in the sympathetic fight-or-flight response mode, enters into the calm and healing state of the parasympathetic system. Then, the people can pay attention and differentiate movements to learn the best option for making them*.	(K) Dance, neuroscience, somatics (H) Aesthetic environment (F) Calmness
Next, I ask him, in a sitting position, to turn his head to the left and then back, noticing which other parts of his body are moving, even minimally. Then, I ask him to move his left shoulder backwards at the same time as he turns his head to the left. I first demonstrate this movement alone, then do it with him, and finally let him do it by himself, with and without my hand on his scapula to help him feel the movement. The sequence continues with the addition of a rotation of the torso to the left and finally the inclusion of the pelvis.	*Starting to move on the functional side of the body is typical of the FM so that the primary motor cortex, responsible for movements, has a functional experience, a brain map of the movement that might be recruited when moving or visualizing the movement on the plegic side* [24]*. Also typical of the FM is the progression that consists of gradually including all the body parts that participate in turning. I thought that increasing the range of motion of the entire body toward the left would help overcome lack of movement in the spastic side. However, it is atypical for the FM to demonstrate the movement. I was progressively shifting from a somatic approach to a dance pedagogy.*	(K) Neuroscience, somatics (H) Reflective touch, active and progressive movement, verbal instruction, visual demonstration (F) Range of motion, body part coordination
At first, I guide these movements verbally and gently with my hands, so that Mr. K can become aware of the movement of the vertebrae in the areas I mention. I ask if he can feel the movement under my fingers. Without hesitation, he answers “yes”. After a few repetitions, I ask him to add a circle (circumduction) with his left arm, keeping eye contact with his hand as his whole body turns to the left. Using my voice, I set a rhythm that I feel matches his capacities. When my hands feel the transmission of movement from one region to another, or my eyes note the fluidity of the movement, I ask, “Do you feel the movement here in this region?” He answers “yes” with a smile as his speech improves from day to day.	*For Moshe Feldenkrais, sensory motor learning requires self-perception within the course of action, including sensitivity to the differentiated segments one at a time before integrating them into a whole. My precise but gentle touch was providing him with feedback to help develop his awareness. My action then showed an appropriate distribution of movement throughout his whole body without too much emphasis at one place, which is one of the aims of the FM* [36].	(K) Somatics (H) Inductive and reflective touch, active and progressive movement, repetition, rhythm, verbal instruction and questioning, visual demonstration (F) Range of motion, quality of movement
We then repeat each step on his plegic side. When he is unable to execute the movement as such in space, I invite him to visualize it slowly. At one point, when he is trying to visualize his right arm circling to the right while actually doing the head, torso, and pelvis movements, he starts laughing and says, “Oops, I was only doing my head”. I start to laugh also, saying, “You’re quite frank, thank you”.	*Visualizing a movement is used in both the FM and dance since it engages the same motor and sensory engrams that are involved in actually performing the movement* [38]*. What strikes me is how Mr. K trusts my approach to overcome movement restrictions by not forcing or pushing, which complements the work of the physiotherapists. I admire him for his open-mindedness.*	(K) Somatics, dance, neuroscience (H) Visualization, effortless (R) Open-mindedness, trust
I then put on some Western music (his partner had told me he likes this type of music) and ask him to perform the movements (head, torso, pelvis, and arms) alternately on the left and right sides, visualizing them on the plegic arm. After a few repetitions, I notice that he is getting into the swing of things, as he is kicking his feet. This prompts me to say, “Now you improvise. You do what you want to move: your head, shoulder, torso, pelvis, arms, from one side to the other”. He begins to move to the rhythm of the music, his foot beating time. When he moves his left arm, the impulse is transmitted to his right side, which doesn’t move as such, but somehow comes alive in resonance with the left side. His partner, the two intern physiotherapists also present, and I are amazed and delighted. We end the session with this amazing moment, as if the movement of the left side combined with the visualization of the plegic side brought life to the plegic side in a way that was palpable for everyone present. Afterwards, one of the physiotherapists says to me, “Music changes everything. It’s what makes it work”.	*Bearing in mind the hope that this project will continue, I wish it had been the two full-time physiotherapists working at the hospital who had witnessed this scene rather than two physiotherapist interns. All the same, during the weekly meeting with the whole treatment team a few days later, the interns mentioned the great cooperation and complementarity we had. The music certainly helped, but it was also the combination of the atmosphere created at the beginning of the class, the progression of the movements chosen, the somatic pedagogy, the quality of the human relationship and the invitation to dance freely without judgement that generated this result. Jarrett* [39]*, reporting a SIMP in a stroke rehabilitation context, mentions “the significance of being able to feel free when confined to a hospital bound by rules and regulations” (p. 21).*	(K) Somatics (H) Aesthetic environment, rhythm, visualization, active and progressive movement, repetition, verbal instruction (R) Open-mindedness, collaboration (F) Body part coordination, quality of movement, motivation
On our way back to his bedroom, I ask him what work he did before retirement. Helen tells me that he had been a boxer and had a boxing school. I exclaim, “That explains everything; you already know how all the joints move together”. He laughed as if he’d played a trick on me. I left, telling him that his boxing was helping his rehabilitation.	*Sööt and Viskus* [40] *mentioned that a “holistic dance teacher approaches teaching through human aspects by taking the distinctive features of students as human beings into account, and by introducing their personal human plan” (p. 292). For Jarrett* [39]*, the results of SIMP are clear: “Dance raised the energy levels of people who participated, contributed to optimism and hope after the trauma of a stroke, and helped people remember who they were” (p. 18).*	(K) Dance, somatics, stroke (H) Verbal questioning (R) Trust
**Session 11—29 January (40 min)**		
We repeat the progressive rotation of the spine to the music. For each movement stage, I ask him to move slowly to feel what the shoulder, then the torso, and finally the pelvis are doing. I prompt him to listen to the connections within his body. Then, I ask him to take the time to visualize the plegic side stage by stage. Coming back to his functional side, we slowly move to dancing, with me counting the movement first on a slow rhythm of four beats, then on two beats as he gradually becomes able to move faster with ease. He really enjoys accelerating gradually. I had never seen Mr. K moving like that with his pelvis participating.	*I could have used this opportunity to add the movement of the foot to the action of turning. There is so much we could do. I prioritize as we go along based on my observations of Mr. K’s physical and emotional state. Surprisingly, I feel comfortable making quick choices supported by my knowledge, although not always clearly formulated in my mind, because I see Mr. K improving.*	(K) Dance (H) Visualization, rhythm, aesthetic environment, slowness, active and progressive movement (F) Body part coordination, quality of movement, motivation
**Session 12—30 January (60 min)**		
The vascular neurologist tells me that Mr. K is leaving the hospital in three days. I then take a moment to speak with Helen in the hall, while Mr. K is having dinner. She finds our meetings extraordinary. She can see the progress and says her partner likes it, because he’s smiling. When we return to the room, he is upset to learn that he is going to a rehabilitation hospital. He says he has not done anything good since he arrived. This is very surprising for Helen and me. I tell him that the other hospital is better suited to his rehabilitation. Suddenly, he asks Helen insistently for a piece of paper and asks me to write down the activities. He wants homework. I write and demonstrate. He asks me if I will come to the rehabilitation hospital. I say it is impossible because I will be away on a trip, but I will have time to visit him there once before I leave.	*Visiting Mr. K in the rehabilitation hospital was not part of the research as initially planned. Being aware of professional boundaries in health settings, I nevertheless decided to do so because it felt right according to my ethical values. Like Korthagen* [41]*, I question the rationale for segmenting our life domains: “[…] to what end the teacher wants to do his or her work, or even what he or she sees as his or her personal calling” (p. 291). My professional life is, in a way, an outgrowth of my personal life. Values such as relationships are central in both. Mr. K and I had developed mutual respect; I cared for him, and I thought that it could increase his motivation to keep going with his rehabilitation. In the same vein, as argued by Elmslie et al.* [42]*, “Dance harnesses humanity” (p. 1).*	(K) Dance (R) Dialogue, trust (F) Motivation

### 3.2. Mrs. T

Mrs. T was a 73-year-old woman who had suffered a left-sided thalamic hemorrhagic stroke with intraventricular extension. She did not require surgery. She was hemiplegic on the right side of her body (lower and upper limbs), experienced spastic paresis in her left leg, could not speak, had difficulty swallowing, and was drowsy. Her NIHSS was 27 at the first encounter. According to the multidisciplinary team and one of her sons, she was a person who was ‘hard on herself’. She could be vigorous or reactive at times, although she had generally been tired and drowsy since the stroke. I (LB) first met Mrs. T about 72 h after her admission to the hospital. I met her 6 times over 9 days for a total of 3 h 50 min of intervention time (average of 38.33 min/session).

**Table 4:** Mrs. T—narrative based on a selected sub-corpus of sessions.

Session 1—22 January (45 min)		
Immediate and embodied experiences	*Self-critical theoretical reflection*	Themes and categories
She has just been put back to bed, having been placed in a wheelchair earlier today for her first physiotherapy session. Upon my arrival, her breathing seems difficult, and she has a lot of spasms in her left leg. Her toes are clenched, and her left foot is slightly dorsiflexed. Her left heel tends towards her right ankle (adduction), and her knee is slightly bent. I make eye contact with Mrs. T as I introduce myself, but she then closes her eyes for what turns out to be most of the session.	*I wonder if it’s relevant to intervene in this context. Insisting appears to contradict the somatic principle of learning through raising awareness, but on the other hand, in a stroke context, stimulating patients is crucial for their recovery. Our presence at the hospital aligns precisely with the quest for an enriched and stimulating environment for patients* [43]*. I am wondering how to navigate with my somatic posture in this medically oriented environment.*	(K) Neuroscience, somatics, stroke
I start with background music to stimulate the patient. After attempting to wake Mrs. T by gently squeezing one of her hands, which proves ineffective, I stop and observe her attentively. I notice impulsive movements in her left foot and leg, which sometimes result in sudden small flexions at the knee with external rotation. It seems to me that her feet offer an entry point for a ‘conversation’ in motion, to ‘tell’ her left leg to calm down.	*FI is a ‘conversation’* [34] *between the practitioner and the ‘student’ through various qualities of touch (e.g., reflective, inductive, directive). For Tufnell* [7]*, it is a “communication through movement and the body” (p. 105). This type of non-verbal conversation might provide Mrs. T with an opportunity for learning, even though it should normally be based on a conscious exploration of movement.*	(K) Somatics, dance (H) Aesthetic environment
After rubbing my hands together to warm them up and placing them on her feet, I apply gentle pressure to different areas under her feet, one foot at a time, to see if there is a response in the ankle, knee, and hip. The heel-ischium connection is observable on the left side but not on the right. I focus on the position of the left foot and rotate the leg slightly externally (so as to match the knee’s orientation), then I alternate moving the leg inward and outward. After numerous repetitions, slowly her ankle relaxes and her heel aligns more with her leg, although her toes remain clenched. Her breathing slows down.	*Initially disconcerted by Mrs. T’s ‘non-participation,’ I now have the feeling that I have somehow initiated a conversation through movement, guided by the quality of her breathing movement. That said, I’ll check with the vascular neurologist whether it’s medically inadvisable to intervene in such a context, even though I remember hearing her say during a team meeting that patients sometimes need to be woken up so that they don’t sleep all the time. Excessive daytime sleepiness in stroke patients can have neurocognitive, functional, and health impacts* [44].	(K) Stroke, somatics (H) Inductive touch, passive movement, slowness, gentleness (R) Collaboration (F) Tension release, calmness, body part coordination
**Session 2—23 January (15 min)**		
Mrs. T is breathing better, but she is still drowsy, and I am told by a nurse that no therapist has been able to work with her today. My attempts at verbal interaction remain difficult, but I go ahead with the vascular neurologist’s approval. She still moves her left foot and leg involuntarily. I start with movements inspired by the ones she is already making. I go a little further today by gently turning her foot outward to initiate a bending movement at the knee and then the hip. I then press under her foot to bring about knee flexion, with the heel sliding towards the pelvis on the bed. Despite her drowsy state, she seems to participate in the knee-bending movement, allowing me to slide her foot close to her pelvis and then to come back and extend her leg, offering less resistance than yesterday. She occasionally relaxes her foot, and her toes gradually become less clenched under my fingers. I then guide the same type of movements on the right (plegic) side, although this leg responds like a puppet.	*My patience is beginning to bear fruit. A form of trust has been established through the slow and repetitive movements. It is reasonable to assume that Mrs. T is in an altered state of consciousness rather than unconsciousness* [45,46]*. It encourages me to keep going with these typical Feldenkrais movements of the leg since learning seems plausible. Starting tomorrow, I am also going to talk to her a bit during the sessions, as she may be partially conscious despite her drowsiness. Because her left leg sometimes responds in spasms, at times I doubt the relevance of what I am patiently trying to do. I also wonder if too much stimulation can exacerbate the patient’s fatigue* [47]*. I will check with the vascular neurologist and decide to shorten the session for today.*	(K) Neuroscience, somatics, stroke (H) Inductive and supportive touch, passive and progressive movement, slowness, repetition (R) Trust, collaboration (F) Tension release, voluntary movement control
**Session 5—28 January (45 min)**		
Upon my arrival, two physiotherapists are mobilizing Mrs. T to sit on the edge of her bed, with her arms resting on a side table in front of her. I observe them working. The patient’s tendency to flex her left leg in external rotation in bed is also visible in a seated position, even if with less tonus. She constantly brings her left leg to the right to cross it over her lower right leg, with her left knee slightly turned to the left, causing her to tilt to her left side. One of the physiotherapists is firmly attempting to ‘place’ the patient’s foot on the ground in a parallel position. The foot just does not stay there.	*It seems to me to be a habit, no matter whether she is lying or sitting. Even though the neurological accident could explain in part what I am observing, I assume that there may have been such a propensity in the body organization before the stroke, an assumption based on my readings of Feldenkrais* [48] *and Hanna* [49].	(K) Stroke, somatics (R) Collaboration
After ten minutes of unsuccessful work with the drowsy patient, the physiotherapists reposition her in bed before I take over. Having noticed that she reproduced the same body organization in the sitting position as in the lying position, I repeat what I have done in the past sessions: slow, gentle rotations of the left leg, both externally and internally, gradually increasing the range of motion in external rotation to further flex the knee. Today, the patient offers less resistance, and the movement is more accessible. I take this opportunity to gently move her left leg from a flex hip and external hip position (‘frog position’) to position her left foot flat on the bed, with her knee pointing towards the ceiling. It works!	*This seems positive since the physiotherapists were unable to establish contact between her foot and the ground. Every time they released the pressure applied to her foot, it would lift off the ground and float in the air. The FM approach helped me to circumvent the difficulty encountered by the physiotherapists. My observation of her movements—both when she was sitting and lying—enabled me to adjust my intervention on the spur of the moment.*	(K) Somatics (H) Inductive touch, passive and progressive movement, slowness, gentleness (F) Tension release, body part coordination, range of motion
On three occasions during this session, I perceive voluntary controlled movements by Mrs. T with her spastic left leg: (1) a knee flexion after pressing on her left heel, (2) a knee adduction while guiding it towards the ceiling, and (3) a leg extension midway in the frog position, as I am subtly guiding her leg to extend fully.	*She is less passive. She participates at times and is more available in the movements, as I can move her left leg without her contracting her muscles. For Rywerant* [34]*, in FI, a proposed movement “should never startle the pupil but rather emerge easily, out of secure patterns established earlier in life or even reflex reactions of the neuro-motor system. The pupil is expected to allow the movements, if possible, without helping or resisting” (p. 27). The use of somatic pedagogy seems to have defused her parasitic inefficient ‘frog’ leg organization lying down, which did not seem to be the case with the directive and corrective approach of the physiotherapists while she was sitting.*	(K) Somatics (H) Inductive and supportive touch, passive and assisted movement (F) Voluntary movement control over involuntary movement, tension release, quality of movement
**Session 6—29 January (65 min)**		
Mrs. T is still in bed, drowsy. I pick up where I left off yesterday, but proceed even more slowly. The repeated movements contribute to the relaxation of her left leg and its alignment with the hip. Instead of constantly folding her leg into a ‘frog’ position in response to any external stimuli (as observed by the nursing staff and clinicians), she manages to keep it calmer and even extends it herself after folding it. As I speak with the vascular neurologist who came to visit her, Mrs. T opens and fully extends her leg on her own, parallel to the other leg, several times. We are witnessing a first, which moves us. Having noticed that I was using background music with the patient, the vascular neurologist confirmed that Mrs. T’s hemorrhagic stroke had not affected the ‘musical’ area of her brain.	*Up until now, I have been using music more as background, but from now on, I will use it to rhythmically guide movement sequences. Music is not used as such in FM, but combining movement with music will enable me to gradually move towards a dance-like approach. Speaking of approach, it turns out that patience, continuity, and gradual variations are pedagogical keys to unlocking Mrs. T’s learning potential despite her drowsiness.*	(K) Somatics (H) Inductive and supportive touch, passive and active movement, slowness, repetition, aesthetic environment, rhythm (R) Dialogue, collaboration (F) Calmness, body part connection
Staff members have probably noticed changes since they are more curious today, asking questions about my work and what is going on in our sessions. They tell me that music is soothing in the neurology unit.	*I realize that my way of talking and guiding the patient’s movements while she appears to be asleep may seem odd to them, as may my use of music. My sessions are quite different from the usual therapy sessions. Collinson* [5] *reports that SIMP is generally misunderstood by the medical team.*	(K) Neuroscience, somatics (H) Aesthetic environment (R) Curiosity, dialogue

The above narratives show what the intervention consisted of and reveal the range of knowledge we drew on in terms of content and pedagogy to craft a unique intervention at the crossroad of many fields of knowledge. As the thematic analysis revealed, we perceived the value of carefully using our voice, hands, and gaze to focus the patients’ attention on perceiving sensations associated with passive or active movement, including breathing, range of motion, rhythm, and effort. This seemed to us to be intimately linked to the changes we observed in the patients’ functionality, as they enhanced their body awareness. The narratives also illustrate the type of relationships developed within the medical team, as well as with the patients and their relatives. Our results confirm that the intervention was very well received by the medical team, the patients, and their relatives.

## 4. Discussion

This study falls into the field of boundary studies in arts and health, which focus on how different disciplines may preserve their intrinsic nature and professional autonomy [50]. The dance, somatics, Feldenkrais, and stroke literature uses multi-dimensional constructs to refer, one way or another, to the person’s ability to perceive sensory and proprioceptive information related to the movement of their own body and body parts. In this discussion, despite differences in semantics and meanings between words such as soma, body awareness, self-awareness, and self-perception, we invite the readers to consider them relatively synonymous. Different terms are used in the studies we refer to, which are not all carried out in stroke units. Nevertheless, they do provide elements of understanding retrospectively about our logics of action, as well as an opportunity to pinpoint avenues for future research.

Apart from the vascular neurologist, we interacted most with the physiotherapists on the clinical team. Their knowledge base included the typical data-driven health sciences, while our knowledge base evolved, and our intervention was somewhat crafted as we went along. Our knowledge was often tacit [51,52] in the sense that it was not easily articulated, as Feldenkrais himself alluded to in the title of his book *The Elusive Obvious* [53]. Working with SIMP in a stroke unit was definitely not that ‘obvious.’ We dissected our knowledge base, which was derived for the most part from the fields of somatics, dance, and stroke. Schön [54] has shown that in spontaneous and intuitive actions, a good practitioner “is continually engaged in a process of appraisal, probing, modelling, testing, diagnosis or evaluation that he or she can barely describe (p. 89). He speaks of tacit experiential knowledge, which may seem at odds with the technical knowledge valued in evidence-based medicine. According to Biswas [55], technical knowledge and intuition exist in a ‘symbiotic relationship’ as the best available evidence can be utilized only through an individual’s expertise. This is akin to the notion of ‘professional artistry,’ which was shelved in favour of science in the early twentieth century [56], a notion echoing Schön’s work on reflective practice. It is “through reflection, either in the moment or after the moment, [that] the practitioner develops the ‘artistry’ of practice” [56] (p. 890).

In the end, although our work was not primarily evidence-based as in the health sciences nor specifically task-oriented as in physiotherapy, it was complementary by aiming indirectly to restore and improve physical function from a different perspective. While the physiotherapists aimed at improving the patients’ impairments with an emphasis on stretching, strengthening, and balancing, we focused on tension release and fine sensing of oneself moving with optimal effort to improve body awareness. One systematic review and meta-analysis of the use of FM in physiotherapy [57] concluded that the FM has positive therapeutic effects comparable to those of physiotherapy with groups of elderly people, people with back pain, and people with neurodegenerative diseases. The authors point out that the FM enhances participation, which is central for physiotherapists working with the International Classification of Functioning, Disability and Health (ICF) [58]. Cardile et al. [59] address the importance of early and intensive multidisciplinary interventions in regaining body awareness. They developed a rehabilitative motor and cognitive program that a physical therapist provided to two late subacute stroke patients. They found that motor and cognitive improvement was correlated with improvement in body awareness as measured with the Multidimensional Assessment of Interoceptive Awareness (MAIA).

Through our research questions, we sought to identify what content and pedagogy we perceived as helping or hindering the intervention in terms of patients’ functionality. According to Doidge [24], the somatic pedagogical principles of small and slow movement may have a neuromodulation effect on the recovering brain due to the chemicals that are released when a person enters a calm state.

At the end of an FM class, people frequently report feeling relaxed or moving with less exertion. We perceived that this was the case for Mr. K and Mrs. T (as well as other patients who participated in the study). We constantly reassessed our choices in the individual sessions, based, among other things, on the ease of the patient’s breathing as it reflects the state of the parasympathetic nervous system [24]. Studies of different populations confirmed a reduction in stress and anxiety after their involvement with the FM [60]. Brummer et al. [61] investigated whether changes in self-perception in healthy populations could be related to a factual change in muscle tension due to the movements performed and lead to a physically enlarged contact surface with the mat. Their measures of pressure and contact surface confirmed the participants’ subjective self-reports. Cardile et al. [62] studied the importance of recovering body awareness in post-stroke rehabilitation. They concluded that stroke patients develop “a better ability to regulate anxiety and distress by paying mindful attention to bodily sensations” (p. 9). For Stephens and Hillier [60], autonomic regulation underlies the benefits of the FM, but the exact mechanisms still need to be investigated. The work of Crivelli et al. [63] is along these lines, focusing on the effects of the FM, with particular emphasis on the neurofunctional correlates of sensorimotor acuity. Similar studies investigating changes in neural activity in motor areas induced by a sensorimotor intervention are needed for interventions like ours to permeate stroke units.

The issue of sensation that is diminished, altered, or absent is important in neurological conditions as it inhibits the sensorimotor loop that leads to overcoming limitations [64]. Given that one in two people suffers reduced sensation following stroke [65], sensory-based interventions deserve further investigation [66,67]. In this regard, Serrada et al. [68] have shown that sensory-based interventions can “enhance adaptive motor cortical plasticity, indicating a potential mechanism which may mediate recovery” (p. 1) and improve somatosensory function after stroke.

For both Mr. K and Mrs. T, we started our sessions by giving tactile information to the patient’s more functional side. With Mr. K, the sessions progressed from typical IF movements, such as turning the head, to a dynamic form of dancing to music. A passive form of SIMP was better suited to Mrs. T’s impairments, with incremental nuances of what seemed to be the same repetition of pushing the sole of the foot. The literature provides arguments for either passive, active, or hybrid sensory training [66].

While we felt free to be creative in tailoring our sessions, this was sometimes accompanied by doubts. We gained confidence by reflecting daily on our sessions, and in the openness of the hospital environment, which has not always been the case in other studies, where demarcating knowledge often enabled powerful groups to control the resources [69]. While we worked more closely with the physiotherapists, we felt welcomed by the whole neurology team. Like Collinson [5], we realized that there was “little understanding of the skills [we could] bring to the wards” (p. 292). However, our intervention aroused interest and curiosity. The artistic dimension (e.g., music, quality of movement) appealed to the medical team, as well as to patients and their relatives. “Including the arts in health care delivery has been shown to support positive clinical outcomes for patients while also supporting other stakeholders, including health care providers, the patient’s loved ones and the wider community” [70]. What characterizes SIMP is that the approach holds “the potential to meet the language of the medical model with the poetic” [71] (p. 6). However, the main challenge remains the implementation of such practices in a care unit where medical examinations and conventional therapies have high priority. We spent almost 20% extra time waiting for each patient to be available for our sessions. Given the difficulty of scheduling our sessions, a more viable option for developing such practices would be to host practitioners to optimize their time spent on site and enable them to see more patients. If we refer to other studies using SIMP in hospitals, at least one or two days a week would be required to do so [71].

Just as intervention opportunities for artists are expanding outside their usual working environment, researchers are increasingly attempting to delineate artists’ requirements to provide fruitful interventions in terms of content and pedagogy [72]. According to Lehikoinen [73], we need to investigate the characteristics of people who successfully cross boundaries and new boundary-spanning roles within health. In this study, we attempted to gain reflective insights into the process of crafting our intervention at the intersection of different fields of knowledge. We did so by applying ECIT methodology and strongly believe that such insights are essential to describe the logic of action behind an intervention. Hospitals need evidence of the benefits of interventions before implementing them, but benefits are closely linked to the behaviours of artists as professionals providing the interventions [14,16,72,74]. Understanding and improving the process of the interventions determines their benefits to a certain extent, which might, in turn, foster the use of somatic-informed movement and dance in hospitals.

Our qualitative approach by no means undervalues a quantitative approach. Rather, it suggests that, for now, dance and SIMP’s benefits are underexplored and that a greater agency is required to assess what are seen as ‘soft outcomes’ or ‘soft targets’ in hospital settings [71]. Collinson [5] reported that hospitals are generally more “concerned with ‘hard outcomes,’ which rarely capture the effects of embodied practice” (p. 298). As Daykin [50] argues, there is a need for more collaboration between stakeholders to support the development of the arts in relation to the health sector.

With respect to what is valued and assessed in stroke rehabilitation, Cardile et al. [59] conducted a scoping review on body awareness. After analyzing the existing literature, they concluded that it is rarely targeted in rehabilitation programs. When it is regarded as an outcome, most of the time it is “considered only from a ‘physical’ perspective such as improvements in walking, balance, or the movement of specific body parts, rather than from a proprioceptive standpoint” (p. 1). Research is, therefore, needed to support the development of new avenues of intervention likely to foster recovery of body awareness after stroke. Our study could be considered a step in this direction.

### Strengths and Limitations

We selected six patients with various neurological impairments to reflect the heterogeneity of cases admitted to acute inpatient stroke units. They all had different stroke localizations, types, and etiologies, so our exploratory work could not evaluate the impact of our intervention on their precise neurological deficit or side of stroke. However, we were able to show that our approach was feasible and appreciated by all those patients presenting various neurological deficits.

Although we partially described the intervention for only two of them, this sample illustrates possible content and pedagogical strategies for meeting individual patient needs and potential benefits. The enhanced critical incident technique, as well as reporting of the data through narratives, helped reveal the prompt decisions we had to make while constructing and applying (sometimes at the same time) our sessions. In a stroke unit, prompt decision making must be prioritized given the patients’ changing conditions. As Mathieu and Hagelsteen [25] argue, “For practitioners, especially in healthcare, relevance and applicability are essential in responding to immediate and practical problem-solving exigencies regarding cases at hand” (p. 2).

The findings of this exploratory study cannot be generalized as it was conducted with a very limited number of patients. The facts that spontaneous recovery can occur in the first few days following stroke and that, in our study, dance and SIMP were combined with conventional therapies in the stroke unit represent other possible limitations of our study. Future research could focus on exploring the comparative advantages and shortcomings of different dance and somatic-informed movement interventions.

The practitioner-researchers’ involvement in the analysis process is another limitation to be taken into consideration, even though peer debriefing took place to ensure rigour in coding and narrative construction. Different results might have been obtained from other dance and SIMP interventions and by other practitioners. Nevertheless, our results offer relevant information that could provide guidance for the future development of this type of intervention in acute stroke units.

Of course, dance and SIMP interventions raise the issue of the difficulty of standardizing such interventions. Like Hoffmann et al. [75], we believe that complex interventions are challenging to describe and replicate. Considering the current status of research, our standpoint is that dance and SIMP intervention “cannot escape a space of indeterminacy troubling to traditional medical research protocols” [76] (p. 161).

## 5. Conclusions

While acknowledging the importance of methodologies that measure the impacts of dance and somatic-informed movement practice in providing solid justification for these practices, our exploratory study constitutes a necessary step in shedding light on their possible contributions in hospital settings. By identifying the different sources of knowledge on which they can be developed, as well as their underlying logics of action, this study shows how such interventions may be complementary to usual stroke rehabilitation therapies. This kind of innovative proposal nevertheless comes with its challenges. While we encountered mainly logistical issues, other types of potential issues have yet to be investigated. Before multidisciplinary teams can implement dance and somatic-informed movement interventions in stroke units, artists need to be trained to do reflective boundary work in an interdisciplinary manner in medical environments.

## Figures and Tables

**Table 1:** Critical incident—data collection.

1. Help/Hinder	Describe a moment when what I said/did gave me the impression of helping/hindering the patient’s condition. Describe exactly what I did/said in detail, so that someone can visualize the gestures and hear the words.
2. Why	In my perception, what aspects of this intervention help/hinder? Why?
3. Logic of action	What was the logic of action? The rationale or intuition that led me to do what I did and to perceive it as helping/hindering?
4. Context	What is my perception of my integration into the interdisciplinary team? What have I done/said to contribute to my integration?
5. Wish list	What could improve the intervention context? How should the next session unfold/be improved?

**Table 2:** Thematic analysis.

Categories	Themes	Research Key Words
Musicality Expressivity	Dance	Knowledge (K)
Somatic principles Somatic modalities	Somatics	
Cognition/brain Muscular tone Neuroplasticity Parasympathetic system	Neuroscience and Stroke	
Information	Verbal inputs	Help (H)
Instructions		
Questions		
Inductive touch	Tactile inputs	
Reflective touch		
Supportive touch		
Demonstration	Visual inputs	
Eye contact		
Visualization	Imaginary inputs	
Active movement	Movements	
Assisted movement Passive movement		
Effortless movement Gentle movement Progressive movement Repetitive movement Rhythmic movement Slow movement	Movement qualities	
Music Qualitative/affective experience	Aesthetic environment	
Open-mindedness Curiosity Trust Dialogue Collaboration	Interactions with stakeholders	Relationships (R)

## Data Availability

The data presented in this study are available upon request from the corresponding author due to privacy, legal, and ethical reasons.

## Abbreviations

The following abbreviations are used in this manuscript:

ATM	Awareness through movement
FI	Functional integration
FM	Feldenkrais method
SIMP	Somatic-informed movement practice

## Appendix A

**Table A1:** Extracts from the saturation grid illustrating some of the codes, categories, and themes for each research question. Note 1: Extracts from data collected for Mr. K and Mrs. T. Note 2: Mrs. T. was an exception among the 6 participants because we could not do any adapted dance movements with her or code her data in terms of verbal and visual inputs due to her drowsiness. Note 3: Under the ‘Relationships’, a small ‘x’ means that the code applies to the relative (in this example, Mr. K’s spouse).

Codes	Mr. K	Mrs. T	Categories	Themes	Research Key Words
Fostering the letting go of unnecessary muscle contractions	X	X	Somatic principles and modalities	Somatics	Knowledge base used for intervention
Fostering ease vs. effort	X	X			
Fostering gentle movement	X	X			
Fostering slow movement	X	X			
Fostering sensory learning	X	X			
Initiating movement with the functional side	X	X			
Sensory communication through movement/the body	X	X			
Combining music and movement	X		Musicality	Dance	
Fostering one’s own movement	X		Expressivity		
Fostering emotional expression	X				
Inducing rhythm	X		Cognition/Brain	Neuroscience and Stroke	
Directing eye movement	X		Muscular tone		
Stimulating senses	X	X	Neuroplasticity		
Engaging imagination	X				
Enriched environment					
Fostering a calm state	X	X	Parasympathetic system		
Inducing movement by touch	X	X	Inductive touch	Tactile inputs	Helping the intervention
Leading a movement by touch	X	X			
Directing attention to a body part through touch	X	X	Reflective touch		
Reflecting on one’s movement by touch	X				
Assisting movement execution through touch	X	X	Supportive touch		
Describing what we do	X		Information	Verbal inputs	
Explaining why we do something	X				
Instructing what/how to do	X		Instructions		
Asking questions about patients’ sensation/perception	X		Questions		
Showing what/how to do	X		Visual demonstration	Visual inputs	
	Patients (Relative)	Team			
Welcoming new perspectives	X (X), X	X	Open- mindedness	Interactions with stakeholders	Relationships
Welcoming complementarity	X (X)	X			
Asking questions, showing interest	X (X)	X	Curiosity		
Willingness to learn	X, X	X			
Confidence in the practitioner	X (X), X	X	Trust		
Mutual respect	X (X)	X			
